# Single-cell analyses identify bioengineered niches for enhanced maintenance of hematopoietic stem cells

**DOI:** 10.1038/s41467-017-00291-3

**Published:** 2017-08-09

**Authors:** Aline Roch, Sonja Giger, Mukul Girotra, Vasco Campos, Nicola Vannini, Olaia Naveiras, Samy Gobaa, Matthias P. Lutolf

**Affiliations:** 1 Institute of Bioengineering, School of Life Sciences and School of Engineering, Ecole Polytechnique Fédérale de Lausanne (EPFL), CH-1015 Lausanne, Switzerland; 2 Department of Medicine, Centre Hospitaler Universitaire Vaudois (CHUV), CH-1015 Lausanne, Switzerland; 3 Institute of Chemical Sciences and Engineering, School of Basic Sciences, EPFL, CH-1015 Lausanne, Switzerland

## Abstract

The in vitro expansion of long-term hematopoietic stem cells (HSCs) remains a substantial challenge, largely because of our limited understanding of the mechanisms that control HSC fate choices. Using single-cell multigene expression analysis and time-lapse microscopy, here we define gene expression signatures and cell cycle hallmarks of murine HSCs and the earliest multipotent progenitors (MPPs), and analyze systematically single HSC fate choices in culture. Our analysis revealed twelve differentially expressed genes marking the quiescent HSC state, including four genes encoding cell–cell interaction signals in the niche. Under basal culture conditions, most HSCs rapidly commit to become early MPPs. In contrast, when we present ligands of the identified niche components such as JamC or Esam within artificial niches, HSC cycling is reduced and long-term multipotency in vivo is maintained. Our approach to bioengineer artificial niches should be useful in other stem cell systems.

## Abstract (web summary)

Haematopoietic stem cell (HSC) self-renewal is not sufficiently understood to recapitulate in vitro. Here, the authors generate gene signature and cell cycle hallmarks of single murine HSCs, and use identified endothelial receptors Esam and JamC as substrates to enhance HSC growth in engineered niches.

## Introduction

The maintenance and regeneration of the blood system relies on a pool of rare hematopoietic stem cells (HSCs) in the bone marrow. These long-lived and mostly quiescent cells can self-renew and give rise to several populations of highly proliferative multipotent progenitors (MPPs) that ensure a constant supply of mature blood cells throughout life. HSCs have been extensively exploited in human medicine for the treatment of hematological and immune diseases. Despite the success of these treatments, the limited number of HSCs available for transplantations still poses a major obstacle for the wider application of HSC-based therapies^1^. Thus, the efficient expansion of HSCs in vitro remains a major goal in the field^2^.

Previous efforts to expand HSCs have largely focused on identifying cytokines or small molecules that target signaling pathways regulating HSC function^3–7^. Such protocols have in some cases demonstrated extensive cell expansion, but single-cell analyses have revealed a concomitant loss of long-term in vivo function of cultured cells after only two or three rounds of cell division^8–10^. The absence of sustained HSC self-renewal might be related to the lack of integration of the multiple signaling components that make up the HSC microenvironment in the native bone marrow. HSC expansion entails the stimulation of proliferation while blocking differentiation, which may be difficult to achieve by targeting only a single microenvironmental parameter^11^. Indeed, HSCs reside in complex and still relatively poorly defined niches^2, 11–19^ that provide a large array of biochemical and biophysical signals that are crucial to maintain the long-term ability of stem cells to self-renew and to give rise to committed progeny. MPPs on the other hand have presumably lost close physical contact to the niche which results in their rapid loss of long-term self-renewal.

In the current work, we aim at bioengineering artificial HSC niches whose design is guided by a systematic analysis of the earliest HSC fate choices occurring during in vitro culture. To this end, we use a combination of single-cell multigene expression analysis and time-lapse microscopy in order to first define gene expression signatures and cell cycle hallmarks of single murine HSC and early MPP. Our analysis revealed 12 differentially expressed genes marking the HSC state, including four genes encoding cell–cell interaction signals in the niche. In particular, we identify two candidate niche interaction ligands, the adherence junction components Esam and JamC that were specifically expressed on primary HSCs, as well as on multiple niche cell populations. Single-cell analyses of dividing HSCs, cultured under serum-free maintenance conditions, reveal a progressive switch from the HSC state to early MPPs with increasing numbers of cell divisions. Strikingly, when we engineer artificial niches to display Esam and JamC, we are able to maintain a rare population of slowly dividing HSCs in vitro. Transplantation of HSCs cultured in these artificial niches resulted in long-term blood reconstitution in vivo. These experiments provide an approach to identify stem cell niche signals based on single-cell fate analysis.

## Results

### Cell-state-specific gene expression signatures

**Fig. 1:**
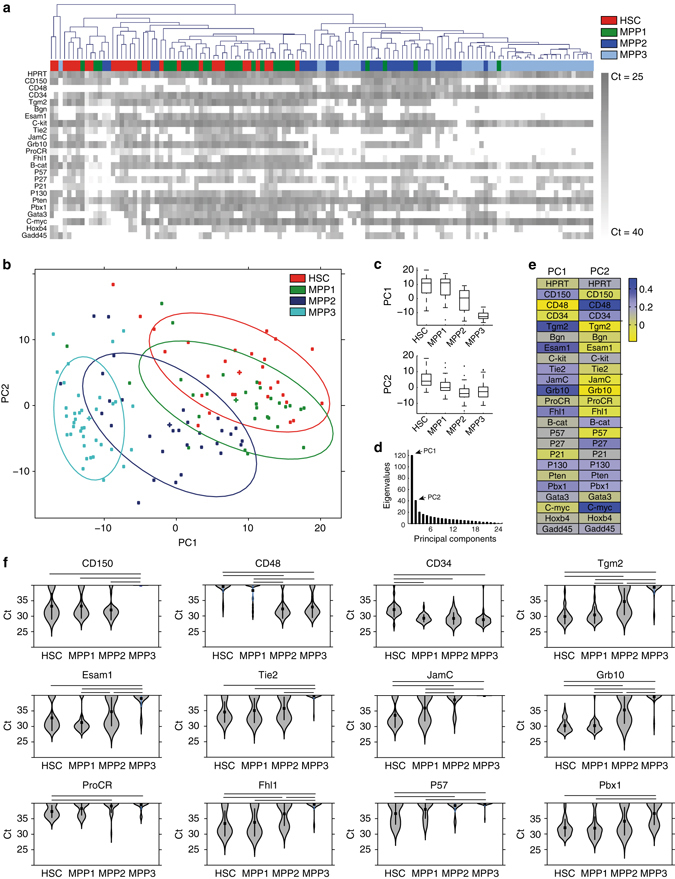
Identification of a stem cell-state-specific gene expression pattern. **a** Heat-map of expression of 24 genes (Ct values) for freshly isolated single HSCs and MPPs. Expression values combined and clustered for HSC, MPP1, MPP2, and MPP3 cells (Number of single cells for HSC *n* = 28, MPP1 *n* = 28, MPP2 *n* = 31, MPP3 *n* = 37, total *n* = 124). *Colored squares* indicate the population of origin for each single cell (*red*, HSC; *green*, MPP1; *blue*, MPP2; *turquoise*, MPP3). **b** Gene expression values of freshly isolated single HSCs and MPPs after principal component analysis (PCA). Individual cells are projected on the first two principal components PC1 and PC2. *Colored points* belong to the indicated populations (*red*, HSC; *green*, MPP1; *blue*, MPP2; *turquoise*, MPP3). Assuming Gaussian distributions, *ellipses* show the 75% regions of highest density. **c** PC1 and PC2 coefficients for all single cells are shown on boxplots. *Center lines* indicate mean. *Box limits* indicate twenty-fifth percentile and seventy-fifth percentile. *Whiskers* indicate extreme data points excluding outliers. *Crosses* indicate outliers. **d** Eigen values of the 24 principal components, indicating the contribution level of principal components PC1 and PC2 selected for the two-dimensional analysis. **e** Heat-map of the coefficients of PC1 and PC2 vectors, corresponding to the weights attributed to each gene by each principal component. **f** Violin plots showing the distribution of gene expression levels for 12 selected genes between HSC, MPP1, MPP2 and MPP3. Genes selected based on ANOVA on HSCs and MPPs (*p* ≤ 0.01). *Squares* indicate the mean. *Vertical lines* indicate the standard deviation. *Gray* areas indicate Kernel probability density. *Horizontal lines* indicate significance in *t*-test pairwise comparisons with *p* ≤ 0.01. See also Supplementary Fig. 1

To characterize the gene expression signature specific to the HSC or MPP state, we performed multigene single-cell expression analyses on freshly isolated long-term HSCs (Lin− C−kit + Sca-1 + CD150 + CD48 − CD34−, termed HSC here) and three closely related MPP populations in the mouse hematopoietic system based on commonly used markers (Supplementary Fig. 1A). We selected 24 candidate genes listed in Supplementary Table 1, which are known markers of the HSC to MPP transition based on microarray studies at the population level^20, 21^ (see Methods). Gene expression levels of all 24 genes were measured for each single cell by multiplex single-cell RT-qPCR. We found marked changes in gene expression profiles among the four populations (Supplementary Fig. 1B). Interestingly, the distribution of gene expression among single cells appears bimodal in most cases, suggesting that gene expression is regulated in an on/off manner, and highlights the importance of studying expression at the single-cell level (Fig. 1f). The bimodal distribution also confirms strong heterogeneity in the HSC compartment, as previously shown by others^22, 23^.

To establish cell-state-specific gene expression patterns, we performed an unbiased hierarchical clustering analysis. Gene expression values for the 124 single cells analyzed were pooled independently of the population of origin of the cells. Hierarchical clustering mostly segregated the cells according to the populations of origin (Fig. 1a). In particular the MPP2 and MPP3 populations formed distinct clusters, whereas the HSC population partially overlapped with the MPP1 population.

The obtained HCL classification was further confirmed by performing principal component analysis (PCA) on the entire gene expression data set. In Fig. 1b, the expression values of single cells for all 24 genes of the four populations are represented in a two-dimensional space composed of the principal component (PC) vectors PC1 and PC2. PC1 and PC2 explained 38.7% and 13.9% of the observed variance, respectively (Fig. 1d). The projection of the expression patterns onto PC1 and PC2 separates individual cells of each population into four clusters (*red*, *green*, *blue* and *turquoise*) representing the 75% regions of highest density (Fig. 1b). HSCs and MPP populations show a gradual reduction of PC1 and PC2 coefficients, suggesting a hierarchical relationship between these populations. PCA reveals that PC1 alone is not sufficient to discriminate between HSC and MPP1 (Fig. 1c, *top panel*) but clearly explains the gene expression differences between MPP populations. The PC1 vector assigns the highest weight to *Tgm2*, *Esam1*, *Fhl1*, and *Grb10* (Fig. 1e), indicating that the expression of these genes is strongly contrasted between early and late MMP stages and hinting to their involvement in the retention of multipotency in early progenitors. On the other hand, PC2 discriminates between HSC and MPP1 but not between MPP populations (Fig. 1c, *bottom panel*). The PC2 vector assigns most weight to CD48 and c-myc as well as to some cell cycle regulatory genes (Fig. 1e). This is consistent with previous findings on c-myc, showing that low c-myc level is associated with maintenance of self-renewal of long-term HSCs^24^. An ANOVA analysis on the four populations identified 12 differentially expressed genes (*p* < 0.01; *Tgm2*, *Esam*, *JamC*, *Tie2*, *ProCR*, *Grb10*, *Fhl1*, *p57*, *Pbx1*, *CD48*, *CD150*, and *CD34*, Fig. 1f). The upregulation of CD48 and CD34 and downregulation of CD150 on MPPs correlate with the phenotypes isolated by FACS.

With gene expression analysis at the single-cell level, we are able to define a specific signature that discriminates HSCs from their closely related multipotent progenitors.

### Identification of proliferation patterns of single HSCs

**Fig. 2:**
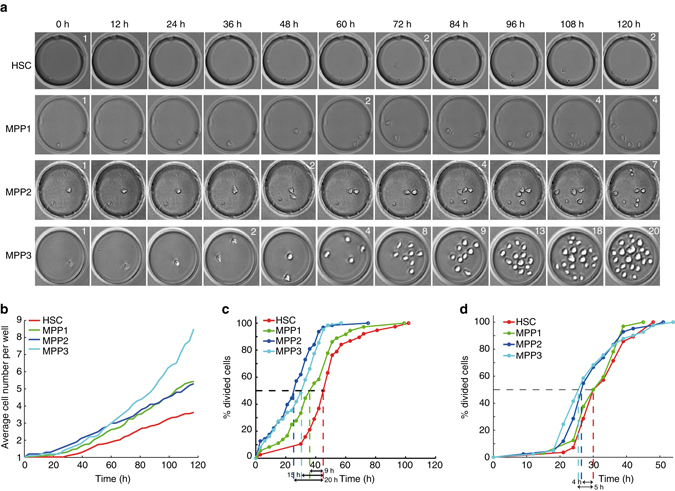
HSCs have a slower division kinetics compared to MPPs. **a** Representative images from time-lapse microscopy showing proliferation of single HSC, MPP1, MPP2 or MPP3 in 100 µm-wide microwells. *White numbers* indicate number of cells in the microwell. **b** Average proliferation of indicated populations (*red*, HSC; *green*, MPP1; *blue*, MPP2; *turquoise*, MPP3) for single cells of each population cultured for 120 h in microwells. Dead cells excluded (Percentage dead cells, HSC 48.7%, MPP1 27.4%, MPP2, 28.5%, MPP3 48.5%). **c** Cumulative histogram of time until first division for indicated populations (*red*, HSC; *green*, MPP1; *blue*, MPP2; *turquoise*, MPP3). **d** Cumulative histogram of time between first and second division for indicated populations (*red*, HSC; *green*, MPP1; *blue*, MPP2; *turquoise*, MPP3). Number of live single cells for HSC *n* = 40, MPP1 *n* = 37, MPP2 *n* = 60, MPP3 *n* = 53. See also Supplementary Fig. 2 and Supplementary Movies 1–4

To associate the specific gene expression signatures with distinct phenotypes of in vitro-cultured cells, we focused on differences in division kinetics of single cells of the four populations, which we hypothesized to be directly linked to the timing of exit from quiescence. Using a previously established high-throughput clonal cell culture system, a microwell array^25, 26^, we analyzed by time-lapse microscopy approximately 50 single cells of each population over 5 days in culture in basal conditions^25^. The cell division patterns of single cells were observed to be strikingly different between the four populations (Fig. 2a and Supplementary Movies 1–4 **)**. On average, HSC divide 1.5-fold less often compared to MPP1 and MPP2, and 2.3-fold less compared to MPP3 (Fig. 2b). The time until the first division is on average 45 h for HSCs, whereas it is reduced by up to 20 h for some MPPs (Fig. 2c). Furthermore, the time difference between the first and the second division is 4–5 hours shorter for MPP2 and MPP3 compared to HSC and MPP1 (Fig. 2d). The decreased cell cycle lengths of HSCs after the first division suggests that they acquire a division pattern resembling that of MPPs.

To relate single-cell division kinetics to specific cell cycle phases, we quantified the DNA content (Hoechst) and Ki67 expression (i.e., proliferating cells) levels of each population by flow cytometry. In line with previous studies^27, 28^ these experiments show that the majority of HSCs (66.5 ± 6.2% in this study) reside in a quiescent G_0_ phase (2n DNA, Ki67^low^), whereas MPP1 and MPP2 show a two- and eight-fold decrease in the proportion of cells in G_0_, respectively (Supplementary Fig. 2A, B). MPP3 show only a threefold decrease of G_0_ cells compared to HSCs, probably due to the presence of quiescent early lymphoid progenitors^29^. Interestingly, the increased proportion of activating cells is proportional to the assessed gene expression level of p57, an important regulator of HSC quiescence^30, 31^, which gradually decreases from 57% in HSCs to only 3% in the MPP3 state (Supplementary Fig. 2C).

### Combined gene expression and cell proliferation analysis

**Fig. 3:**
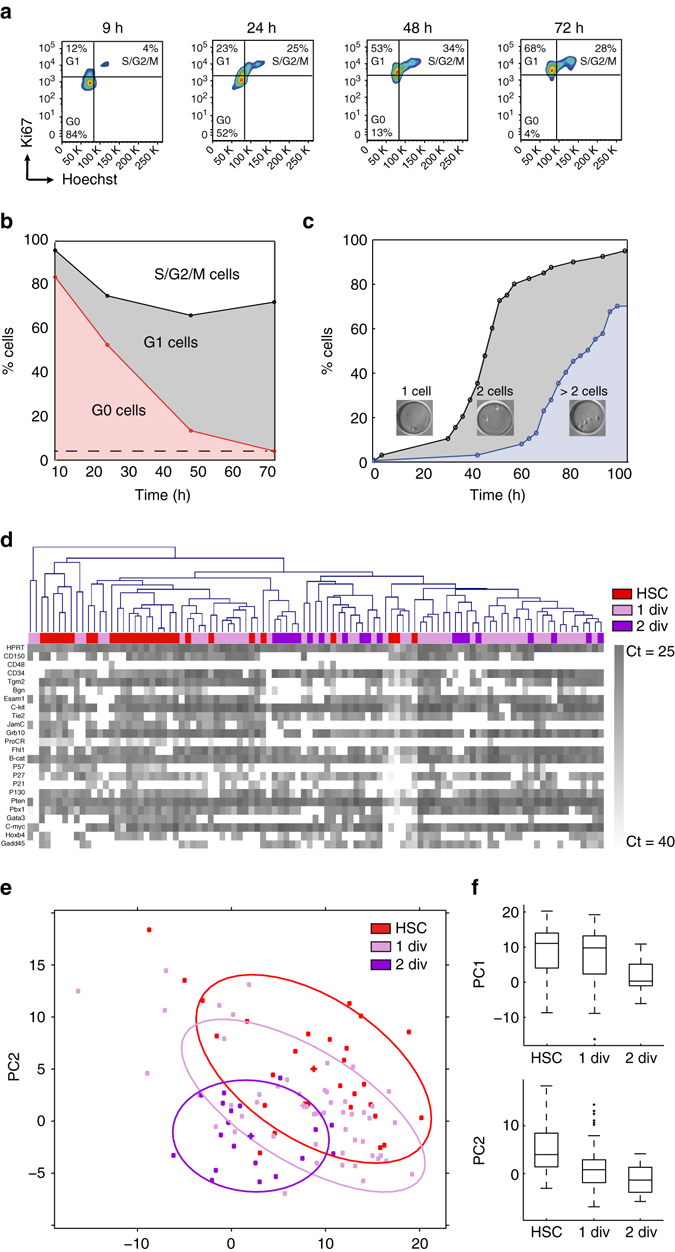
HSCs cultured in vitro lose their stemness upon cell division and acquire an MPP-like gene expression profile. **a** Flow cytometry plots showing the percentage of cells in the G_0_, G_1_, or S/G_2_/M phases of the cell cycle based on DNA content (Hoechst) and Ki67 expression for HSCs cultured for 9, 24, 48, or 72 h. **b** Percentage of cells in G0 (*red*), G1 (*gray*) or S/G2/M (*white*) after 9, 24, 48, or 72 h in culture. **c** Percentage of cells having undergone no division (*white*), one division (*gray*), or more than one division (*blue*) in function of time. **d** Combined heat-map of Ct values for 24 genes from freshly isolated HSCs and cultured HSCs after hierarchical clustering (Number of single cells for HSC *n* = 28, 1 div *n* = 52, 2 div *n* = 19, total *n* = 99). *Colored squares* indicate the population of origin for each single cell (*red*, freshly isolated HSC; *mauve*, one division in culture; *purple*, two divisions in culture). **e** Single-gene expression values of cultured HSCs compared to freshly isolated HSCs after principal component analysis (PCA). Individual cells are projected on principal components PC1 and PC2 conserved as in Fig. 1b. Colored points belong to the indicated populations (*red*, freshly isolated HSC; *mauve*, one division in culture; *purple*, two divisions in culture). Assuming Gaussian distributions, ellipses show the 75% regions of highest density. **f** PC1 and PC2 coefficients for all single cells are shown on boxplots. *Center lines* indicate mean. *Box limits* indicate twenty-fifth percentile and seventy-fifth percentile. *Whiskers* indicate extreme data points excluding outliers. *Crosses* indicate outliers. See also Supplementary Figs. 3 and 4

To better understand the observed HSC fate choices that occur in vitro, we performed single-cell gene expression analyses of HSCs that underwent a defined number of divisions. We performed time-lapse microscopy in combination with micromanipulation to pick cell progeny with a defined growth history, distinguishing between HSC daughters that were generated by one or two cell divisions. To determine the optimal time point to collect HSC progeny from microwell cultures, we first determined changes in cell cycle phase during 3 days in culture (Fig. 3a, b). Quiescent HSCs slowly enter G_1_, nearly linearly changing from ca. 84% of cells being in G_0_ after 9 h to ca. 4% after 72 h. In comparison, time-lapse microscopy shows that after 72 h ca. 85% of all HSCs have divided once and ca. 25% of all cells have divided twice (Fig. 3c). We therefore picked HSC clones by micromanipulation after 70 h in microwell culture.

We analyzed the single-cell gene expression patterns of cultured cells after one or two divisions and compared them to the gene expression pattern of freshly isolated HSCs (Fig. 3d–f). Using both hierarchical clustering (Fig. 3d) and principal component analysis based on vector PC1 and PC2 described above for freshly isolated HSs (Fig. 1b and e), we showed that increasing numbers of cell divisions corresponds to the observed changes in gene expression between freshly isolated stem cells and progenitor cells of increasing commitment. Cells that divided once showed a very heterogeneous pattern, partially overlapping with freshly isolated HSCs, whereas cells that divided twice acquired a MPP-like gene expression pattern (Fig. 3d and 3e, Supplementary Fig. 3) suggesting that these cells could have lost their stemness after two divisions. This gradual loss of stemness is most evident when looking specifically at PC1 scores, which are maintained at the same level for HSCs undergoing one division in vitro compared to freshly isolated HSCs (Fig. 3f, *top panel*), whereas they are decreased when cells undergo two divisions. On the other hand, PC2 scores representing long-term HSC maintenance/self-renewal genes such as c-myc are lower for both populations of cultured cells compared to freshly isolated HSCs, independently of the division history (Fig. 3f, *bottom panel*).

Combined time-lapse microscopy and micromanipulation allows the tracking of related daughter cells after division of a single HSC. The difference between two cells of a pair can be calculated using the Euclidian distance between the two cells based on the 24 genes (Symmetry Index or SI). Intriguingly, cell pairs with a high SI value generally have one cell clustering together with freshly isolated HSCs and the other one with more differentiated cells. In contrast, pairs with a low SI value mostly cluster together on the side of differentiated cells. Similarly, cells that have undergone two divisions are generally clustering together and never in the HSC cluster (Supplementary Fig. 4B). It is tempting to speculate that these differences between paired daughter cells could be linked to different HSCs fate choices, i.e., asymmetric (self-renewal) versus symmetric (self-renewal and commitment) cell divisions.

The strategy utilized here constitutes an interesting way to assess the fate of stem cell in culture based on in vitro readouts, by associating expression of specific stemness genes with the mitotic activity of the cells. This tool can be used as a preliminary screen for the effect of different culture conditions on the stem cell maintenance, before confirmation by state-of-the-art in vivo assays. In this particular case, HSCs were cultured under basal conditions with limited added growth factors. In this condition, we show that HSCs gradually acquire a transcriptional profile that is similar to that of MPPs, where only a small fraction of cells after the first round of division maintains high expression of certain genes specifically expressed in the HSC compartment. Culturing HSCs in an environment mimicking the in vivo niche is hypothesized to be important for the maintenance of stemness. Thus we aimed at creating an artificial niche, composed of known HSC/niche interaction ligands, and using the tool we developed to assess stem cell maintenance based on in vitro readouts.

### Common expression of receptors on niche cells and HSCs

**Fig. 4:**
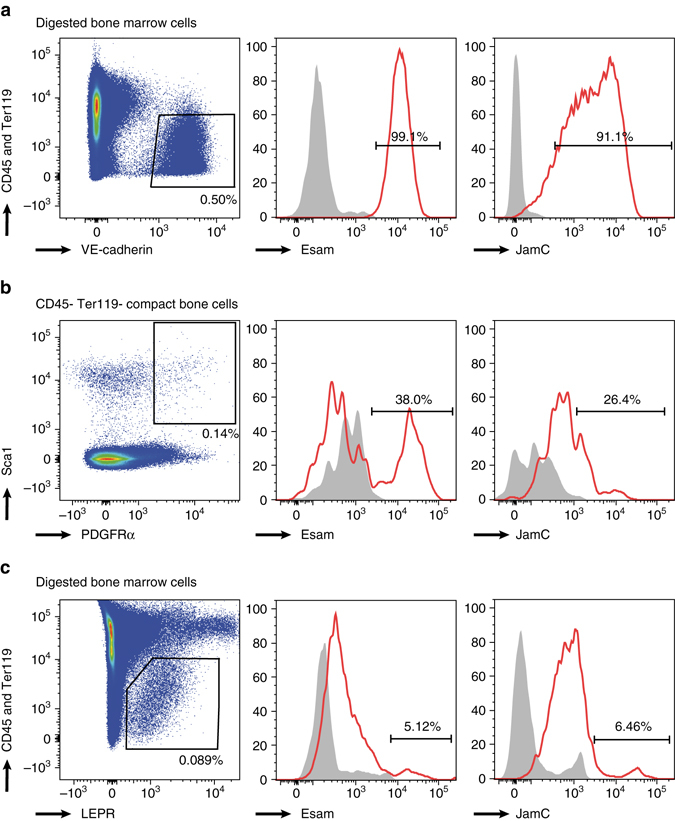
Esam and JamC are expressed on endothelial, mesenchymal, and perivascular niche cells. Flow cytometry plots showing in *red* the percentage of Esam or JamC expression on **a** VE-Cad + CD45-Ter-119- digested bone marrow cells, or **b** PDGFRα + Sca-1 + CD45-Ter-119- digested compact bone cells, or **c** LEPR + CD45-Ter-119- digested bone marrow cells. *Filled gray histograms* show FMO (fluorescence minus one) controls for Esam and JamC. See also Supplementary Fig. 5

To understand the cell–cell interaction and adhesion of HSCs with niche cells, we selected 4 membrane-bound receptors out of our set of factors specifically expressed by HSCs, namely Tie2^32^, ProCR^33^ (encoding the EPCR protein), JamC^34, 35^, and Esam^36^ (Fig. 1f). To confirm specific expression of these receptors on HSCs at the protein level, we stained cells of the HSC and MPP populations with antibodies against these antigens and analyzed them by flow cytometry (Supplementary Fig. 5A). The vast majority of HSCs were positive for these markers; 98% were EPCR-positive, 99% Esam-positive, 83% Tie2-positive, and 85% JamC-positive (Supplementary Fig. 5B). Conversely, for the most committed MPPs, the number of positive cells drops to 57% for EPCR, 17% for Esam, 8% for Tie2, and 50% for JamC (Supplementary Fig. 5B). The loss of surface expression of EPCR, Esam and JamC was gradual from the HSC to the MPP3, in agreement with our single-cell gene expression measurements. Tie2 is to some extent an exception to this trend, with a surprisingly high percentage of expression (80%) in the MPP2 population. Nevertheless, these results suggest a key function for the interaction of HSCs with niche cells via the identified receptors. Indeed, Angiopoietin-1, the ligand for Tie2, is know to be expressed by osteoblast in the endosteal niche^32^. On the other hand, relatively little is known about the expression patterns of JamC or Esam on HSC niche cells. Both markers are expressed on endothelial cells at tight junctions^15^. Endothelial cells have been shown to play an important role in the maintenance of HSCs in the in vivo niche^14^. We thus investigated the expression of both markers on VE-Cadherin-expressing bone marrow endothelial cells^14, 37^ and found expression of JamC and Esam on the majority of VE-Cad + cells (Fig. 4a **)**. Interestingly, expression of JamC and Esam was also detected on other HSC-supportive niche cell types, namely the LEPR + perivascular stromal cells^14, 38^, as well as the PαS (PDGFRα + Sca-1+) mesenchymal stem cells^39–41^, with expression of Esam on up to 40% of PαS mesenchymal stem cells (Fig. 4b and c).

These results suggest a key role for adhesion receptors Esam and JamC in the maintenance of HSCs in the in vivo niche, by directly mediating the interactions between HSCs and niche cells. Thus we hypothesized that the rapid loss of the stem cell gene expression patterns measured under basal culture conditions (Fig. 3d–f) would be caused by an absence of critical regulatory signals from an instructive niche.

### Artificial niches preserve stemness

**Fig. 5:**
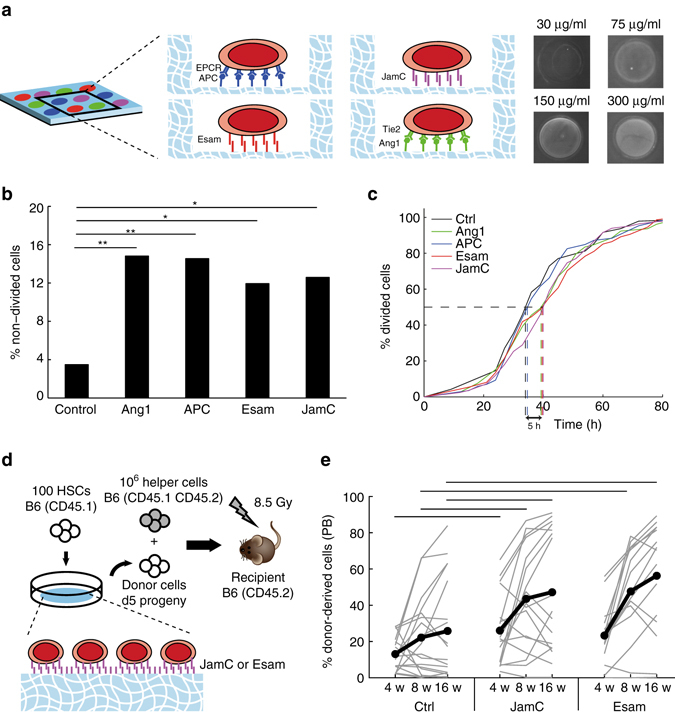
HSCs exposed to an artificial in vitro niche functionalized with cell-cell interaction molecules slow down proliferation and retain in vivo reconstitution potential. **a** PEG microwell array functionalized with APC, JamC, Esam, or Ang1. Immunofluorescent images of JamC-functionalized microwells stained with anti-JamC antibody and fluorescently conjugated anti-rat antibody. **b** Percentage of cells undergoing no divisions over 120 h in non-functionalized microwells (control) or microwells functionalized with APC, Ang1, Esam or JamC. Graph indicates percentages of cells. Dead cells excluded. *p ≤ 0.05, **p ≤ 0.01 in Fisher’s exact test. **c** Cumulative histogram of time until first division for HSC cultured in non-functionalized microwells (control, *black*) or microwells functionalized with APC (*blue*), Ang1 (*green*), Esam (*red*) or JamC (*purple*). Number of single cells is for control *n* = 86, for Ang1 *n* = 162, for APC *n* = 165, for Esam *n* = 134, for JamC *n* = 143. **d** HSCs were cultured for 120 h in non-functionalized microwells (control) or microwells functionalized with JamC or Esam and the progeny of 100 cells was recovered and injected in lethally irradiated mice. Blood of recipient mice was analyzed at 4, 8, or 16 weeks. **e** Graph indicates percentage of donor-derived cells at 4 weeks, 8 weeks, or 16 weeks in mice engrafted with cells exposed to non-functionalized microwells (control) or microwells functionalized with JamC or Esam. *Graph* represents pooled data from two independent experiments. *Gray lines* indicate percentage of donor-derived cells for single mice of both experiments. *Black line* indicates mean for all animals. Number of recipients surviving at 16 weeks for both experiments is *n* = 18 for control, *n* = 17 for JamC, and *n* = 15 for Esam. *p ≤ 0.05, **p ≤ 0.01. *Bars* indicate significance at *p* ≤ 0.05 in non-parametric Mann–Whitney test. See also Supplementary Figs. 6–8

In order to mimic cell–cell and cell-ECM interactions in the niche, we employed a recently developed micro-engineering approach^42^ to display ligands to the previously identified niche components. Hydrogel microwells were chemically modified with each of the four candidate niche components Ang1, APC, Esam and JamC, the ligands for Tie2, EPCR, Esam and JamC (Fig. 5a). Single HSCs exposed to these artificial niches markedly changed their mitotic activity (Fig. 5b and c): the percentage of non-dividing HSCs over 5 days increased by 4-fold when exposed to Ang1 and APC, and by 3.5-fold with JamC and Esam (Fig. 5b **)**. For the 50% of HSCs that divided, we also measured a delay of 5 hours before undergoing a first cell division for cells in the presence of Ang1, JamC and Esam (Fig. 5c). In order to correlate these changes in single-cell growth kinetics to specific changes in the cell cycle phase, we assessed for each cell population the DNA content by Hoechst staining after 70 h in culture (Supplementary Fig. 6A). This analysis shows substantially reduced proportions of G_2_/M cells (4n DNA) and correspondingly increased G_0_/G_1_ (2n DNA) fractions, namely 20% for Ang1, around 30% for APC and Esam, and up to 55% for JamC (Supplementary Fig. 6B). Therefore, the identified exogenous niche candidates slow down entry of HSCs into the cell cycle when tethered to a hydrogel substrate. Notably, cells exposed to the control condition were all exposed to protein A, which did not lead to the changes observed in presence of the candidate niche components, excluding the presence of an off-target effect due to cell adhesion to the substrate. This suggests that these factors might be components of HSC niches that participate in HSC maintenance in vivo by reducing their mitotic activity. Of note, exposure of HSCs to JamB, the heterotypic ligand for JamC^34, 35^, also led to an increase of non-dividing HSCs, and a decrease in the proportion of cycling, but to a smaller extent compared to JamC (Supplementary Fig. 7A, B).

The ultimate proof that HSCs have retained their stem cell potential is their ability to repopulate the blood system in vivo in a lethally irradiated mouse. To assess the presence of functional stem cells within the pool of cultured cells, we performed in vivo blood repopulation assays according to the scheme shown in Fig. 5d. Based on the above in vitro cell cycle and proliferation kinetics data (Fig. 5b and c, Supplementary Fig. 6A, B), we selected JamC and Esam for further in vivo repopulation assays. Mice injected with HSC progeny cultured 5 days under control conditions showed a very heterogenous engraftment, reflecting the heterogeneity of gene expression patterns of dividing HSCs that showed maintenance of a stem cell-like phenotype only for a minority of cells (Fig. 5e). As a result a majority of animals showed engraftment below 40% at 16 weeks (*n* = 13 of 18) (Fig. 5e, Supplementary Fig. 8A). In contrast, mice injected with cells cultured on JamC-modified or Esam-modified artificial niches showed improved engraftment levels (Fig. 5e, Supplementary Fig. 8A). JamC exposure led to an increased number of highly reconstituted animals (*n* = 8 of 17 above 60%) but a retained heterogeneity of engraftment, and seemingly a myeloid-biased engraftment pattern (Fig. 5e, Supplementary Fig. 8A). Esam exposure led a more consistent and robust engraftment with a engraftment levels mostly above 60% (*n* = 10 of 17) (Fig. 5e, Supplementary Fig. 8A).

These results were also confirmed in a secondary transplantation, where no reconstitution activity was observed in secondary recipients from engrafted primary recipient bone marrow for the control group. In contrast, engrafted primary recipient bone marrow for the JamC and Esam groups showed reconstitution in secondary recipients above 10% at 16 weeks (Supplementary Fig. 8B).

These results show that, when grown on hydrogel substrates functionalized with putative niche ligands, some HSCs maintain their long-term stem cell potential in contrast to standard cell culture conditions. Indeed, a single factor like Esam allows a reduction of heterogeneity in the engraftment potential of cultured HSC, suggesting that more complex artificial niches composed of multiple factors might allow a more consistent maintenance of HSCs for multiple days in vitro.

## Discussion

The in vitro maintenance and expansion of HSCs without genetic manipulation remains a major challenge in bone marrow regenerative medicine. To tackle this challenge, a better understanding of the mechanisms that control the earliest fate choices of HSCs in their niche is key. However, mechanistic studies are hindered by a lack of markers and tractable single-cell assays to clearly distinguish between HSC self-renewal and differentiation fate choices. Here, we employed a combination of single-cell approaches to define distinct gene expression signatures of the stem and progenitor cell states and applied this knowledge to systematically analyze fate choices that occur in vitro.

Single-cell gene expression analyses have previously been used to investigate cell fate choices in the hematopoietic^43–45^ and in other stem cell systems^46, 47^. For example, erythroid differentiation was investigated using the immortalized EML hematopoietic cell line, showing a stochastic distribution of molecular programs coordinating the transition between self-renewal and commitment^45^. More recently, distinctive transcription factor expression states were identified between HSCs and myeloid and lymphoid progenitors, identifying transcription factor networks involved in controlling HSC fate choices^22–44^. However, such an approach has not been used to probe hematopoietic stem cell fate choices in culture.

In this study, we identified a single-cell-state-specific gene expression pattern of HSCs, which we showed to be drastically altered after the first division of HSCs in vitro. With regard to this result, we hypothesized that the cell cycle kinetics influences the state of HSCs in vitro. It is well known that the HSC micorenvironment within the native bone marrow present important factors to maintain self-renewing HSCs. The lack of these signals might be related to the rapid loss of stemness in vitro.

In order to validate the presented concept, four known cell surface receptors, Tie2, JamC, Esam and EPCR, that influence the maintenance of HSCs in vivo, were selected (Figs 1f, 4, and Supplementary Fig. 5). The receptor tyrosine kinase Tie2 is a well-established HSC niche receptor^32^, and the interaction with its ligand Ang1 is known to regulate HSCs primarily via control of quiescence. The junctional adhesion molecule JamC has been shown to be expressed on the surface of HSCs^34, 35, 48^ and, in association with proteins such as Par-3, Par-6 and aPKC, regulates cell polarity in tight junction complexes such as those involved in spermatid polarization^49^ or leukocyte-endothelium interactions^15^. Accordingly, JamC-deficient mice show no changes in the HSC pool size but rather an increase in the number of myeloid progenitors^48^. In contrast, the heterotypical interaction of JamC with JamB is important for the maintenance of HSCs, since JamB-deficient mice have a decreased pool of quiescent HSCs^34^. Intriguingly, the Par-6/aPKC polarity complex has been suggested to play a role in regulating asymmetric polarization of HSCs^50^. Esam also belongs to the family of junction adhesion molecules and is expressed at the surface of LKS Thy1.1^+^ Flt3^−^HSCs^36^. LKS Esam^high^ cells have enhanced repopulating capacity compared to Esam^low^ cells^36^. Esam-deficient mice show a defect in hematopoiesis only under stress induced via 5-FU treatment^51^. EPCR, encoded by the *ProCR* gene, has been reported to be expressed by HSCs of the CD34- SP (side population) phenotype^33^, but its role in HSC regulation remains to be elucidated.

Our study confirmed the expression of the selected factors on HSCs and thereby suggests the presence of niche cells in the bone marrow that express the complementary ligands. Indeed, our experiments show that the endothelial receptors Esam and JamC are expressed on bone marrow endothelial cells^14^, as well as other niche cell types including LEPR + perivascular cells^14, 38^ and PαS mesenchymal stem cells^39–41^. It should be interesting to further identify and characterize the niche cells to which HSCs possibly bind via adherens junction, and to assess the phenotypic and functional overlap with other possible niche cells in the bone marrow such as Cxcl12-abundant reticular (CAR) cells^18^, Nestin-positive mesenchymal stem cells^16^ or TGFβ-secreting Schwann cells^19^.

We bioengineered novel HSC culture substrates to display the corresponding ligands of the four selected niche markers and thereby influencing the state of HSCs in vitro. The artificial niches were able to slow down cell cycling of single cells. Our single-cell gene expression analysis of HSCs proposes that low dividing HSCs maintain an HSC-like profile compared to highly dividing cells that acquire MPP-like profiles. Together, our results suggest that slowing down cell cycle allows the maintenance of the stem cell identity in culture.

Of note, HSC cultured in the presence of tethered factors were observed to constantly move around on the substrate, irrespective of whether they divided or not (not shown). This would rule out the anchoring of quiescent HSCs in an adherens junction-like manner as is likely to happen in a stem cell niche in vivo^52, 53^. More complex artificial niches mimicking cell–cell junctions, most likely composed of more than one cell–cell interaction protein and higher protein concentrations have to be engineered. This also opens the possibility to study cell division symmetry by using engineered microenvironments. It is possible to envision that by providing a complex array of localized signaling cues, such microenvironments could induce cell polarity and asymmetric segregation of cell determinants, leading to asymmetric cell divisions.

## Methods

### Mice

All animal experiments were performed in compliance with the Swiss law after approval from the local (Service Vétérinaire de l’Etat de Vaud) and federal authorities (VD 2135.3a/ETV 26747, VD 2242.2/ETV 28153). C57Bl/6J and C57Bl/6J Ly5.1 female and male mice, in the age of 8–12 weeks, were purchased from the Charles River Laboratories International and maintained at the Center for Studying Living System (CAV) at the EPFL in microisolator cages. Mice were provided continuously with sterile food, water, and bedding.

### Flow cytometry and cell sorting

Hematopoietic stem and progenitor cells were isolated from flushed bone marrow of 8–12 week C57Bl/6 mice. Erythroid cells were eliminated by incubation with red blood cell lysis buffer (Biolegend). Lineage depletion (CD3, B220, Ter-119, CD11, Gr-1) was performed using the Hematopoietic Progenitor Cell Enrichment set (BD Biosciences). Cells were stained with SAV-PETxRed (1:200, Life Technologies), Kit-PECY7 (1:200, 2B8, BioLegend), Sca-1-APC (1:100, D7, BioLegend) or –PerCPCY5.5 (1:200, E13-161.7, BioLegend), CD150-PE or –PECY5 (1:100, TC15-12F12.2, BioLegend), CD48-PB (1:100) or-PE (1:1’000) (HM48-1, BioLegend), CD34-FITC or-eFluor660 (1:25, RAM34, eBioscence). Hematopoietic stem and progenitor cells were sorted on the Lin- Kit + Sca-1 + (KLS) population based on CD150, CD48 and CD34 expression. For the analysis of Esam, EPCR, JamC, and Tie2 expression, hematopoietic stem and progenitor cells were stained with either Esam-PE (1:100, 1G8, BioLegend), CD202-PE (1:100, 1560, eBioscence), anti-JamC (1:100, CRAM18 F26, Abcam) combined with anti-rat IgG conjugated to Alexa546 (1:1’000), or biotinyalated anti-CD202b (1:200, TEK4, eBioscience) combined with SAV-PETxRed (1:200, Life Technologies) and Lin-APC antibody cocktail (1:100, BD Biosciences). For the analysis of mesenchymal stem cells, flushed bones were finely cut and digested for 1 h at 37 °C in Collagenase type I (Life Technologies)^40, 41^. PαS (PDGFRα + Sca-1+) mesenchymal stem cells were stained with Sca-1-BV711 (1:150, D7, BD Biosciences), and PDGFRα-APC (1:100, APA5, BioLegend). For the analysis of perivascular niche cells and endothelial cells, bone marrow was flushed and digested for 15 min in Collagenase type IV (Gibco), Dispase (Gibco), and DNAse (Roche)^14, 37^. Cells collected from digested bone marrow were blocked for 5 min with FBS and human IgG (Privagen). Perivascular cells were stained with anti-LEPR (R&D) combined to anti-goat IgG conjugated to AlexaFluor488. All three cell types were stained with Esam-PE (1:100, 1G8, BioLegend), or anti-JamC (1:100, CRAM18 F26, Abcam) combined to anti-rat IgG conjugated to PECy7 (1:200, Poly4054, Biolegend), and with CD45-biotin (1:200, 30-F11, eBioscience), Ter-119-biotin (1:200, TER-119, eBioscience), and Streptavidin-AlexaFluor488 or 647 (1:1000, Life Technologies). For the analysis of endothelial cells, VE-Cadherin staining was performed in vivo by IV injection of 10 µg VE-Cadherin eFluor660 (eBioBV13, eBioscience) 10 min before killing the animals^14, 37^.

### Gene selection for RT-qPCR

A total of 24 candidate genes listed in Supplementary Table 1 were chosen, including 10 genes whose expression was found to be selectively enriched in HSCs (phenotype: Thy1.1lo/Flk2- LKS cells) compared to MPPs^21^ under steady-state hematopoiesis, as well as in non-mobilized and non-leukemic HSCs^20^. These ten genes comprise Bgn encoding biglycan, an ECM proteoglycan, Tgm2, an enzyme mediating crosslinking of ECM proteins, Esam and JamC (also termed Jam3), glycoproteins localized at intercellular junctions mediating cell–cell adhesion and cell polarity (JamC), Tie2 and ProCR, genes encoding for extracellular receptors, the intracellular adapter molecules Grb10 and Fhl1, the cyclin-dependent kinase inhibitor 1C (p57, Kip2) and the oncogene Pbx1. We also included three components of the cell cycle machinery, p27, p21 and p130, that were reported to be critical for maintenance of quiescent HSCs^54^, and six genes important for HSC maintenance (*b-Cat* ^55^, *Pten* ^56^, *Gata3* ^57^), self-renewal (*Hoxb4* ^58^, *c-Myc* ^24^), and stress response (*Gadd45* ^59^). Finally, we included CD150, CD48, and CD34, the phenotypic markers used for cell isolation.

### Single-cell RT-qPCR

Real-time quantitative PCR (RT-qPCR)was performed with Gene Expression TaqMan Assays. For analysis of freshly isolated cells, single cells were sorted in the wells of a 96-well PCR plate (BioRad) containing 10 μl of lysis solution. For analysis of cultured cells, micromanipulated single cells were expelled into 0.2 ml PCR tubes containing 10 μl of lysis solution. Lysis solution consisted of 9 μl single-cell lysis solution supplemented with 1 μl single-cell DNAse I (Single Cell-to-Ct Kit, Life Technologies). Cells were incubated in the lysis solution at room temperature for up to 30 min. One microliter of single-cell stop solution was added to the samples and incubated at room temperature up to 20 min. Lysed cell samples were kept on ice up to 2 h and then stored at −20 °C. Reverse transcription and pre-amplification were performed sequentially in the lysed cell sample using Single Cell-to-Ct Kit (Life Technologies). Conditions for reverse transcription were 10 min at 25 °C, 60 min at 42 °C, and 5 min at 42 °C. Twenty-four Gene Expression TaqMan Assays (Life Technologies, Supplementary Table 1) were pooled and diluted at 0.2× in 1× TE buffer pH 8.0 for pre-amplification. Samples were incubated for 10 min at 95 °C and pre-amplified for 14 cycles of 15 s at 95 °C and 4 min at 60 °C. The pre-amplified samples were diluted 1:20 in 1× TE buffer pH 8.0 and stored at −20 °C. RT-qPCR was performed with Gene Expression TaqMan Assays (Life Technologies, Supplementary Table 1) on a 7900HT system (Applied BioSystems). Conditions for amplification were 2 min at 50 °C and 10 min at 94.5 °C followed by 40 cycles of 5 s at 97 °C and 1 min at 59.7 °C.

Samples that did not express HPRT were excluded from analysis but Ct values were not normalized to HPRT expression, as the expression of housekeeping genes is very variable when assessing expression at the single-cell level. A minimum of 30 single cells were analyzed for each population. Expression values over the threshold of the machine (Ct = 40) were set to 40.

Technical controls for our single-cell gene expression assays were performed as shown in Supplementary Fig. 9. We lysed 10 single LKS CD150- cells and split the lysate into 10 equal volumes, each representing a ‘single-cell equivalent’ containing the same genetic material, thus ruling out biological variability. We performed RT-qPCR for HPRT expression on the samples to assess technical variability of the assay. Ct values obtained for 10 single-cell equivalents spread with a standard deviation of ca. 0.5 Ct. In contrast, the Ct values obtained for 45 single LKS CD150- cells that were lysed independently have a much higher variability, with a standard deviation of ca. 1.8 Ct. The variability observed can thus be accounted for a biological heterogeneity.

### Cell cycle analysis

Cell cycle analysis was performed on FACS-sorted cells or on cells recovered from 96-well flat-bottom microplates (BD) after culture on flat hydrogels. Cells were fixed and permeabilized using Cytofix/Cytoperm kit (BD Biosciences). Cells were labeled with Ki67 FITC (BD Bioscences) overnight and Hoechst 33342 (Life Technologies) for 10 min.

### Hematopoietic stem cell culture

Hematopoietic stem or progenitor cells were cultured under sterile conditions in serum-free media (Stemline II Hematopoietic Stem Cell Expansion Medium, Sigma) supplemented with 100 ng/ml SCF and 2 ng/ml Flt3 ligand in 5% CO_2_ at 37 °C. One thousand cells were seeded per well of a 4-well plate (Nunc) in 4 ml of medium. The plate was transferred to the microscope and incubated for 30 min to let individual cells randomly sediment at the bottom of the microwells. Cells were cultured and imaged for up to 5 days on the microscope. Alternatively, 300 cells were seeded per well of 96-well flat-bottom microplate (BD) in 200 μl medium and the plate was transferred in the incubator and cultured for up to 5 days.

### Single-cell proliferation analysis

Individual cells cultured in microwells were imaged for up to 5 days on a microscope (Zeiss Axio Observer Z1) equipped with a motorized stage. The stage was programmed to scan the microwell array surface and acquire images at 5× magnification of multiple positions every 3 h. Single-cell proliferation kinetic was assessed based on time-lapse movies. Cells were scored dead when they stopped moving on the microwell surface.

### Micromanipulation of HSC progeny for single-cell analysis

Single cells undergoing 1 or 2 divisions after 70 h were isolated from the microwells by micromanipulation in custom-made microcapillaries of 20 μm diameter (Eppendorf), using TransferMan NK2 and CellTram Vario (Eppendorf). Single cells were expelled from the microcapillaries into 10 μl of lysis solution for subsequent single-cell PCR.

### Generation of artificial niches

A DNA spotter equipped with solid pins was used to dispense PEG-conjugated proteins or Fc-chimeric proteins, dissolved in 30% glycerol, on micropillars of a microfabricated silicon stamp. Micropillars had a dimension of 450 μm in diameter, and 100 μm in height. Thin layers of hydrogel were formed at the bottom of 4-well plates (Nunc) by crosslinking 4-arm-PEG-thiol (PEG-SH, 10 kDa) with 8-arm-PEG-vinylsulfones (PEG-VS, 10 kDa) at 5% w/v with an excess of 9% SH groups. In the case where Fc-chimeric proteins were used, PEG-conjugated protein-A was added to the gel at 86 μg/ml. Hydrogel films were micropatterned by soft embossing with the protein-adsorbed silicon stamps for 1 hour^37^.

Alternatively, for bulk analysis, flat thin layer of hydrogels were formed in 96-well flat-bottom microplates (BD) at 5% w/v with an excess of 9% SH groups, in the presence, or absence, of protein-A. After complete crosslinking of the hydrogel, 40 μl of PEG-conjugated proteins or Fc-chimeric proteins, dissolved in 30% glycerol, were added to cover the whole surface of the gel, and incubated for 1 hour.

### Transplantation

HSCs were isolated from Ly5.1 donors and 100 cells were cultured for 5 days in 96-well plates on flat hydrogels functionalized with JamC or Esam as described above. The progeny of 100 cells were recovered from the hydrogel and directly injected into lethally irradiated CD45.2 recipient mice together with 10^6^ Sca-1-CD150- helper cells from double congenic CD45.1/45.2 mice. Helper cells were obtained through depletion of Sca-1 + and CD150 + cells from whole bone marrow by magnetic cell separation using Sca-1-PE antibody (E13-161.7, Biolegend), CD150-PE antibody (TC15-12F12.2, BioLegend) and anti-PE MicroBeads (Miltenyi Biotec). Recipient mice were bled at 4, 8, and 16 weeks post transplant and peripheral blood was analyzed for donor chimerism by staining red blood cells-depleted samples with CD45.1 FITC (1:200, A20, Biolegend), CD45.2-PB (1:200, 104, Biolegend), CD3-PE (1:200, 17A2, Biolegend), CD19-PE (1:500, 6D5, Biolegend), Gr-1-APC (1:1000, RB6-8C5, Biolegend), and F4/80-APC (1:750, BM8, Biolegend). For secondary transplants, bone marrow was collected from primary recipients (three mice for control group, two mice for JamC group and four mice for Esam group). Bone marrow from each primary recipient was injected into three lethally irradiated CD45.2 secondary recipient mice at 3 × 10^6^ cells per secondary recipient. Secondary recipient mice were bled at 4 and 8 weeks post transplant.

### Statistical analysis

Significant genes were found using one-way ANOVA at a Bonferroni-corrected alpha level of 0.01. Pairwise comparisons were performed using Student’s *t*-test with alpha level of 0.01. Analysis of contingency was performed using Fisher’s exact test with alpha level of 0.05. Hierarchical clustering was performed on single-cell samples using Pearson correlation and average linkage. PCA was performed on mean-centered data using Matlab (The Mathworks Inc, Natick MA, USA). Transplantation results were analyzed using a non-parametric Mann–Whitney test. Kernel density probabilities for violin plots were performed in R (R Foundation for Statistical Computing, Vienna, Austria). Symmetry indexes (SI values) were calculated as the Euclidan distance between two cells of a pair based on the expression of the 24 genes:

$\sqrt {\mathop {\sum}\nolimits_{i = 1}^{24} {{{\left( {{\rm{lo}}{{\rm{g}}_2}\left( {{\rm{C}}{{\rm{t}}_i}\,{\rm{cell}}\,a} \right) - {\rm{lo}}{{\rm{g}}_2}\left( {{\rm{C}}{{\rm{t}}_i}\,{\rm{cell}}\,b} \right)} \right)}^2}} } ,$ (where *i* is the gene number and cell *a* and *b* are two sister cells of a pair).

### Data availability

The authors declare that all data supporting the findings of this study are available within the article and its Supplementary information files or from the corresponding author upon reasonable request.

## Electronic supplementary material

Supplementary Information

Supplementary Movie 1

Supplementary Movie 2

Supplementary Movie 3

Supplementary Movie 4

## References

[1] Brunstein CG (2010). Allogeneic hematopoietic cell transplantation for hematologic malignancy: relative risks and benefits of double umbilical cord blood. Blood.

[2] Kunisaki Y, Frenette PS (2012). The secrets of the bone marrow niche: enigmatic niche brings challenge for HSC expansion. Nat. Med..

[3] Boitano AE (2010). Aryl hydrocarbon receptor antagonists promote the expansion of human hematopoietic stem cells. Science.

[4] Goessling W (2011). Prostaglandin E2 enhances human cord blood stem cell xenotransplants and shows long-term safety in preclinical nonhuman primate transplant models. Cell Stem Cell.

[5] North TE (2007). Prostaglandin E2 regulates vertebrate haematopoietic stem cell homeostasis. Nature.

[6] Sauvageau G, Iscove NN, Humphries RK (2004). In vitro and in vivo expansion of hematopoietic stem cells. Oncogene.

[7] Zhang CC (2006). Angiopoietin-like proteins stimulate ex vivo expansion of hematopoietic stem cells. Nat. Med..

[8] Ema H, Takano H, Sudo K, Nakauchi H (2000). In vitro self-renewal division of hematopoietic stem cells. J. Exp. Med..

[9] Kent DG, Dykstra BJ, Cheyne J, Ma E, Eaves CJ (2008). Steel factor coordinately regulates the molecular signature and biologic function of hematopoietic stem cells. Blood.

[10] Nakauchi H, Sudo K, Ema H (2001). Quantitative assessment of the stem cell self-renewal capacity. Ann. N. Y. Acad. Sci..

[11] Calvi LM (2003). Osteoblastic cells regulate the haematopoietic stem cell niche. Nature.

[12] Chow A (2011). Bone marrow CD169+macrophages promote the retention of hematopoietic stem and progenitor cells in the mesenchymal stem cell niche. J. Exp. Med..

[13] Ding L, Morrison SJ (2013). Haematopoietic stem cells and early lymphoid progenitors occupy distinct bone marrow niches. Nature.

[14] Ding L, Saunders TL, Enikolopov G, Morrison SJ (2012). Endothelial and perivascular cells maintain haematopoietic stem cells. Nature.

[15] Ebnet K, Suzuki A, Ohno S, Vestweber D (2004). Junctional adhesion molecules (JAMs): more molecules with dual functions?. J. Cell. Sci..

[16] Mendez-Ferrer S (2010). Mesenchymal and haematopoietic stem cells form a unique bone marrow niche. Nature.

[17] Nakamura-Ishizu A, Suda T (2013). Hematopoietic stem cell niche: an interplay among a repertoire of multiple functional niches. Biochim. Biophys. Acta.

[18] Sugiyama T, Kohara H, Noda M, Nagasawa T (2006). Maintenance of the hematopoietic stem cell pool by CXCL12-CXCR4 chemokine signaling in bone marrow stromal cell niches. Immunity.

[19] Yamazaki S (2011). Nonmyelinating schwann cells maintain hematopoietic stem cell hibernation in the bone marrow niche. Cell.

[20] Forsberg EC (2010). Molecular signatures of quiescent, mobilized and leukemia-initiating hematopoietic stem cells. PLoS ONE.

[21] Forsberg EC (2005). Differential expression of novel potential regulators in hematopoietic stem cells. PLoS Genet..

[22] Guo G (2013). Mapping cellular hierarchy by single-cell analysis of the cell surface repertoire. Cell Stem Cell.

[23] Wilson NK (2015). Combined single-cell functional and gene expression analysis resolves heterogeneity within stem cell populations. Cell Stem Cell.

[24] Wilson A (2004). c-Myc controls the balance between hematopoietic stem cell self-renewal and differentiation. Genes Dev..

[25] Lutolf MP, Doyonnas R, Havenstrite K, Koleckar K, Blau HM (2009). Perturbation of single hematopoietic stem cell fates in artificial niches. Integr. Biol..

[26] Vannini N (2012). Identification of in vitro HSC fate regulators by differential lipid raft clustering. Cell Cycle.

[27] Arai F, Suda T (2007). Maintenance of quiescent hematopoietic stem cells in the osteoblastic niche. Ann. N. Y. Acad. Sci..

[28] Wilson A (2008). Hematopoietic stem cells reversibly switch from dormancy to self-renewal during homeostasis and repair. Cell.

[29] Pelayo R (2006). Cell cycle quiescence of early lymphoid progenitors in adult bone marrow. Stem Cells.

[30] Matsumoto A (2011). p57 is required for quiescence and maintenance of adult hematopoietic stem cells. Cell Stem Cell.

[31] Zou P (2011). p57(KiP2) and p27(Kip1) cooperate to maintain hematopoietic stem cell quiescence through interactions with Hsc70. Cell Stem Cell.

[32] Arai F (2004). Tie2/angiopoietin-1 signaling regulates hematopoietic stem cell quiescence in the bone marrow niche. Cell.

[33] Balazs AB, Fabian AJ, Esmon CT, Mulligan RC (2006). Endothelial protein C receptor (CD201) explicitly identifies hematopoietic stem cells in murine bone marrow. Blood.

[34] Arcangeli M-L (2011). JAM-B regulates maintenance of hematopoietic stem cells in the bone marrow. Blood.

[35] Arcangeli ML (2014). Function of Jam-B/Jam-C interaction in homing and mobilization of human and mouse hematopoietic stem and progenitor cells. Stem Cells.

[36] Ooi AGL (2009). The adhesion molecule esam1 is a novel hematopoietic stem cell marker. Stem Cells.

[37] Inra CN (2015). A perisinusoidal niche for extramedullary haematopoiesis in the spleen. Nature.

[38] Zhou BO, Yue R, Murphy MM, Peyer JG, Morrison SJ (2014). Leptin-receptor-expressing mesenchymal stromal cells represent the main source of bone formed by adult bone marrow. Cell Stem Cell.

[39] Greenbaum A (2013). CXCL12 in early mesenchymal progenitors is required for haematopoietic stem-cell maintenance. Nature.

[40] Houlihan DD (2012). Isolation of mouse mesenchymal stem cells on the basis of expression of Sca-1 and PDGFR-alpha. Nat. Protoc..

[41] Morikawa S (2009). Prospective identification, isolation, and systemic transplantation of multipotent mesenchymal stem cells in murine bone marrow. J. Exp. Med..

[42] Gobaa S (2011). Artificial niche microarrays for probing single stem cell fate in high throughput. Nat. Methods.

[43] Franco CB, Chen CC, Drukker M, Weissman IL, Galli SJ (2010). Distinguishing mast cell and granulocyte differentiation at the single-cell level. Cell Stem Cell.

[44] Moignard V (2013). Characterization of transcriptional networks in blood stem and progenitor cells using high-throughput single-cell gene expression analysis. Nat. Cell Biol..

[45] Pina C (2012). Inferring rules of lineage commitment in haematopoiesis. Nat. Cell Biol..

[46] Buganim Y (2012). Single-cell expression analyses during cellular reprogramming reveal an early stochastic and a late hierarchic phase. Cell.

[47] Guo G (2010). Resolution of cell fate decisions revealed by single-cell gene expression analysis from zygote to blastocyst. Dev. Cell.

[48] Praetor A (2009). Genetic deletion of JAM-C reveals a role in myeloid progenitor generation. Blood.

[49] Gliki G, Ebnet K, Aurrand-Lions M, Imhof BA, Adams RH (2004). Spermatid differentiation requires the assembly of a cell polarity complex downstream of junctional adhesion molecule-C. Nature.

[50] Hope KJ (2010). An RNAi screen identifies Msi2 and Prox1 as having opposite roles in the regulation of hematopoietic stem cell activity. Cell Stem Cell.

[51] Sudo T (2012). The endothelial antigen ESAM monitors hematopoietic stem cell status between quiescence and self-renewal. J. Immunol..

[52] Arai F, Hirao A, Suda T (2005). Regulation of hematopoietic stem cells by the niche. Trends Cardiovasc. Med..

[53] Zhang J (2003). Identification of the haematopoietic stem cell niche and control of the niche size. Nature.

[54] Passegué E, Wagers AJ, Giuriato S, Anderson WC, Weissman IL (2005). Global analysis of proliferation and cell cycle gene expression in the regulation of hematopoietic stem and progenitor cell fates. J. Exp. Med..

[55] Scheller M (2006). Hematopoietic stem cell and multilineage defects generated by constitutive beta-catenin activation. Nat. Immunol..

[56] Yilmaz OH (2006). Pten dependence distinguishes haematopoietic stem cells from leukaemia-initiating cells. Nature.

[57] Ku C-J, Hosoya T, Maillard I, Engel JD (2012). GATA-3 regulates hematopoietic stem cell maintenance and cell-cycle entry. Blood.

[58] Antonchuk J, Sauvageau G, Humphries RK (2001). HOXB4 overexpression mediates very rapid stem cell regeneration and competitive hematopoietic repopulation. Exp. Hematol..

[59] Liebermann DA, Hoffman B (2007). Gadd45 in the response of hematopoietic cells to genotoxic stress. Blood Cells Mol. Dis..

