# Supplementary material for: Single-cell analyses identify bioengineered niches for enhanced maintenance of hematopoietic stem cells
Source: Nat Commun. 2017 Aug 9;8:221. doi: 10.1038/s41467-017-00291-3 (PMC5548907; doi:10.1038/s41467-017-00291-3)
Supplement: Supplementary file 1 — Supplementary Information [file 41467_2017_291_MOESM1_ESM.pdf]

File Name: Supplementary Information
Description: Supplementary Figures, Supplementary Table.

File Name: Supplementary Movie 1
Description: Single cell time-lapse microscopy of HSCs and MPPs. Representative movies from time-lapse microscopy showing proliferation of single HSC in 100μm-wide microwells.

File Name: Supplementary Movie 2
Description: Single cell time-lapse microscopy of HSCs and MPPs. Representative movies from time-lapse microscopy showing proliferation of single MPP1 in 100μm-wide microwells.

File Name: Supplementary Movie 3
Description: Single cell time-lapse microscopy of HSCs and MPPs. Representative movies from time-lapse microscopy showing proliferation of single MPP2 (3) in 100μm-wide microwells.

File Name: Supplementary Movie 4
Description: Single cell time-lapse microscopy of HSCs and MPPs. Representative movies from time-lapse microscopy showing proliferation of single MPP3 in 100μm-wide microwells.

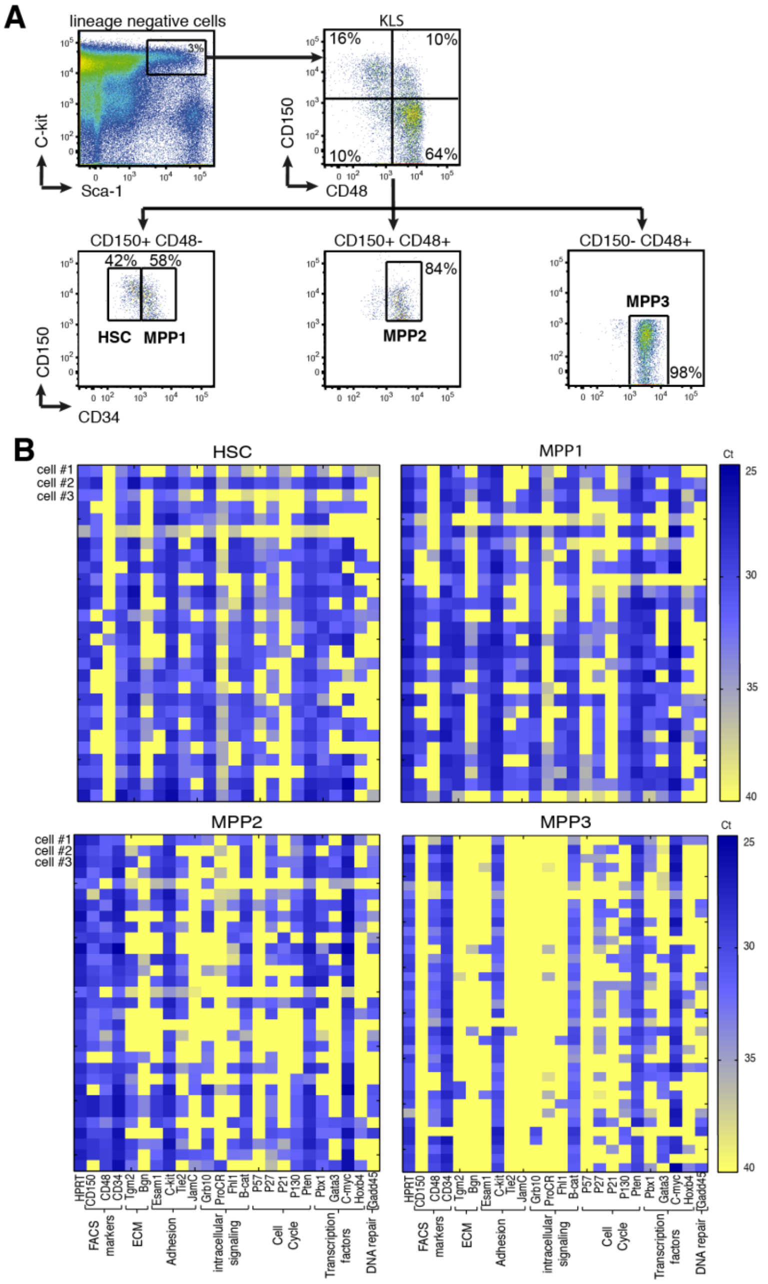

Supplementary Figure 1. Gene expression profile of HSCs vs MPPs. (A) Representative flow cytometry plots showing subdivision of the four studied populations within Lin^{neg} C-Kit^{+} Sca1^{+} (LKS) BM based on the expression of CD150, CD48 and CD34. HSC: CD150+ CD48- CD34-; MPP1: CD150+ CD48- CD34+; MPP2: CD150+ CD48+ CD34+; MPP3: CD150- CD48+ CD34+. (B) Heat-maps of gene expression levels for 24 genes from individual cells of each population. Raw Ct values are shown ranging from blue, highly expressed, to yellow, no detected expression. Number of single cells for HSC n=28, MPP1 n=28, MPP2 n=31, MPP3 n=37.

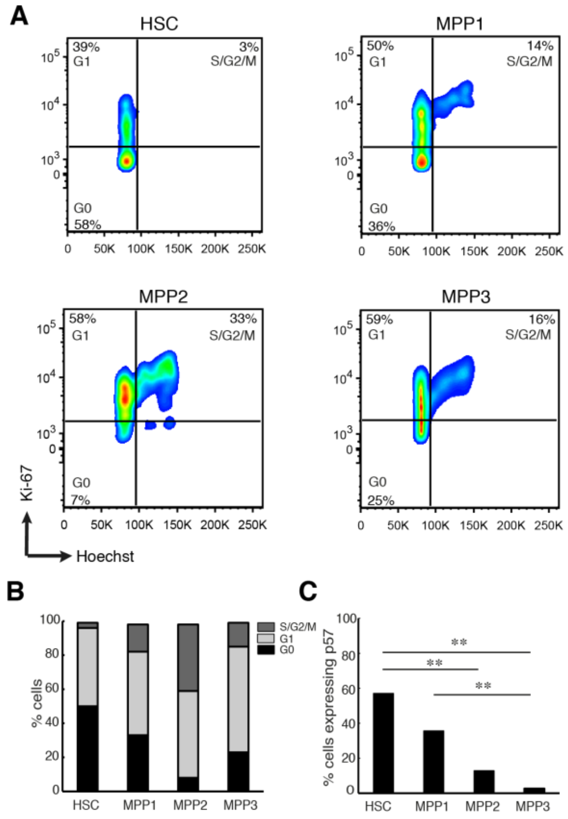

Supplementary Figure 2. Decrease of p57 expression in progenitors compared to HSCs correlates with decrease of quiescence. (A) Flow cytometry plots showing the percentage of cells in the G0, G1, or S/G2/M phases of the cell cycle based on DNA content (Hoechst) and Ki67 expression for HSC, MPP1, MPP2 or MPP3. (B) Percentage of cells in G0 (black), G1 (light grey) or S/G2/M (dark grey) for HSC, MPP1, MPP2 or MMP3. (C) Percentage of single cells expressing p57 (Ct<40) for HSC (n=28), MPP1 (n=28), MPP2 (n=31), or MPP3 (n=37). ** $p \leq 0.01$ in Fisher's exact test.

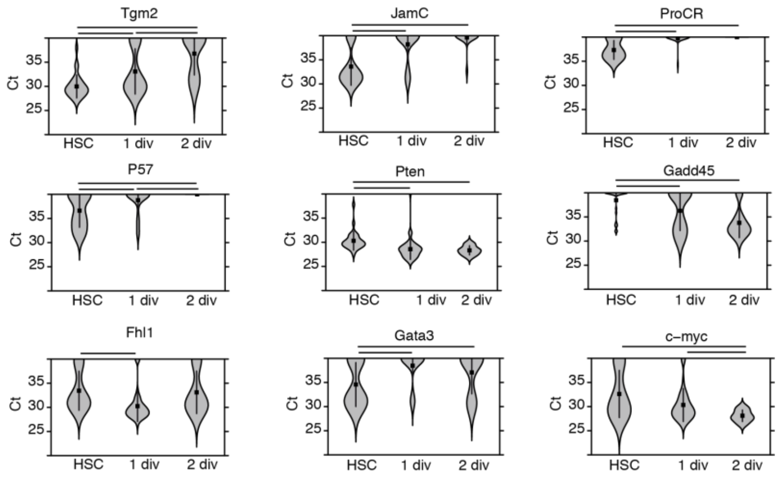

Supplementary Figure 3. Identification of genes differentially expressed between freshly isolated HSCs and cultured HSCs. Violin plots showing the distribution of gene expression levels for the 9 selected genes between freshly isolated HSC, and cultured HSCs. Genes selected based on ANOVA on freshly isolated HSCs and cultured HSCs ($p \leq 0.01$). Squares indicate the mean. Vertical lines indicate the standard deviation. Grey areas indicate Kernel probability density. Horizontal lines indicate significance in t-test pairwise comparisons at $p \leq 0.01$. Number of single cells for HSC n=28, 1 div n=52, 2 div n=19.

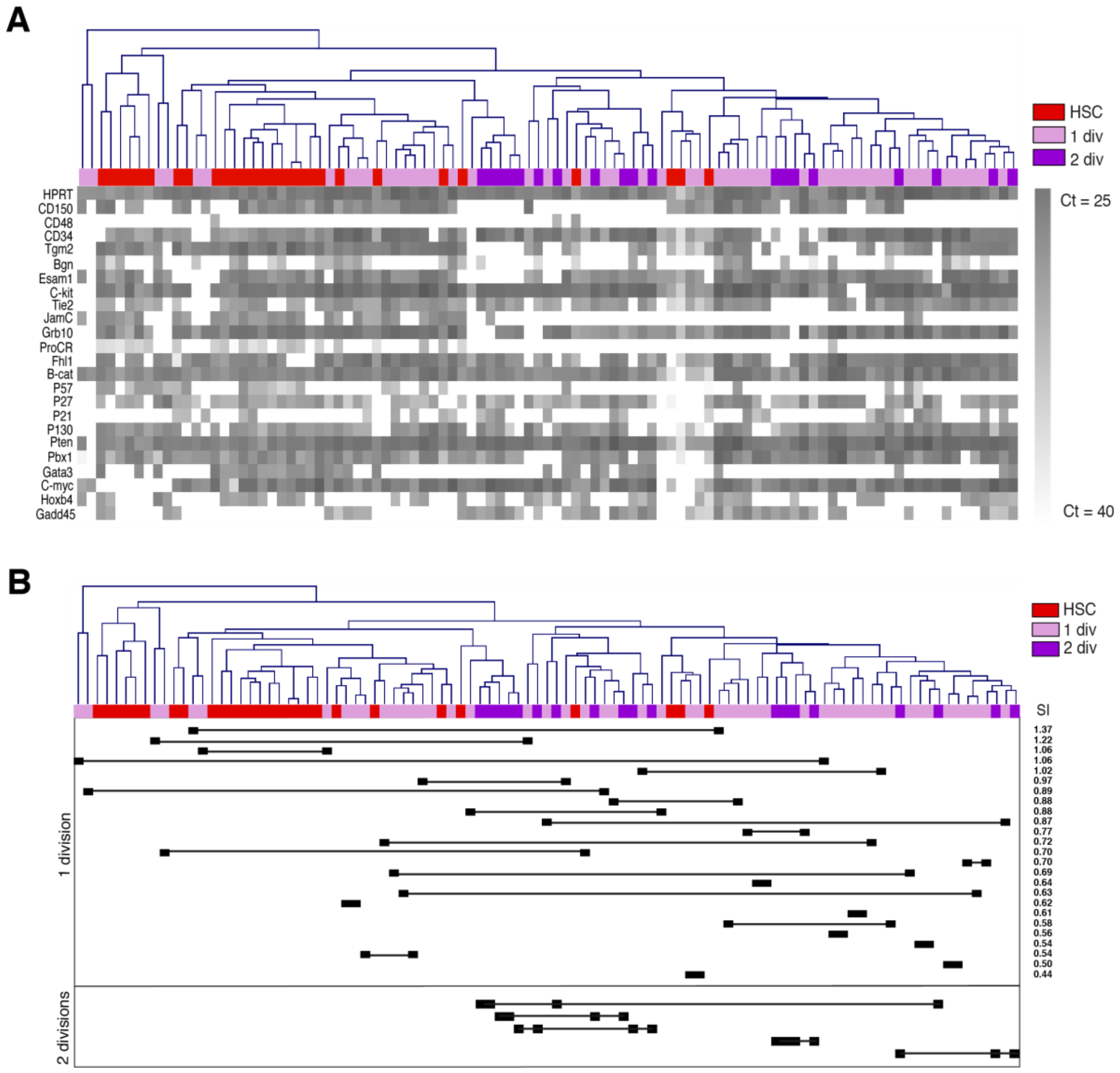

Supplementary Figure 4. Sister cells mostly distribute asymmetrically after one division and symmetrically after two divisions. (A) Clustered heat-map from Figure 3D. Hierarchical clustering of Ct values from freshly isolated HSCs and cultured HSCs. (B) Relationship of paired daughter cells indicated on the clustering map. Connected black squares indicate sister cells generated from one single HSC after one or two divisions. SI values indicate the symmetry index of each pair, calculated based on the expression of the 24 genes using Euclidian distance. A higher SI values indicates a higher distance between sister cells.

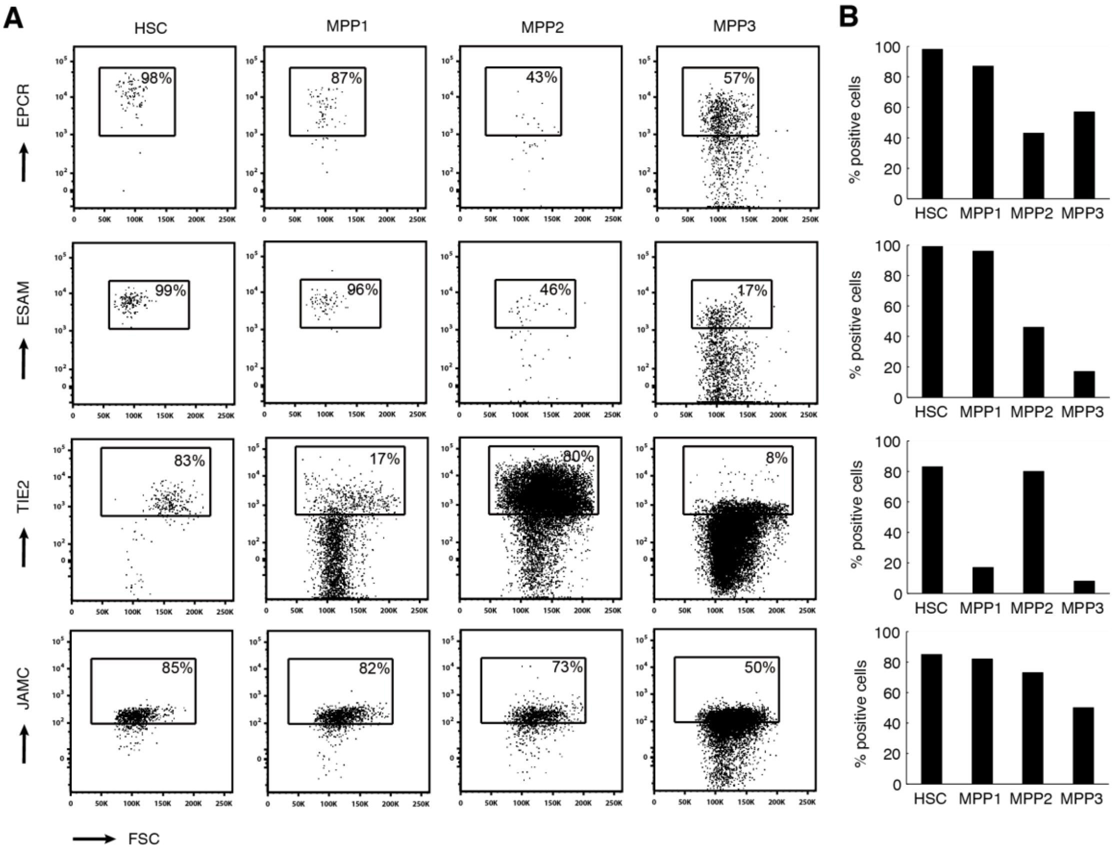

Supplementary Figure 5. Surface expression of EPCR, Esam, Tie2 and JamC is high on HSCs and low on MPPs. (A) Flow cytometry plots showing the expression of EPCR, Esam, Tie2 or JamC in function of forward scatter FSC (percentage positive cells are shown). (B) Percentage of EPCR+, Esam+, Tie2+ or JamC+ cells within populations of HSC, MPP1, MPP2, MPP3 based on flow cytometry analysis.

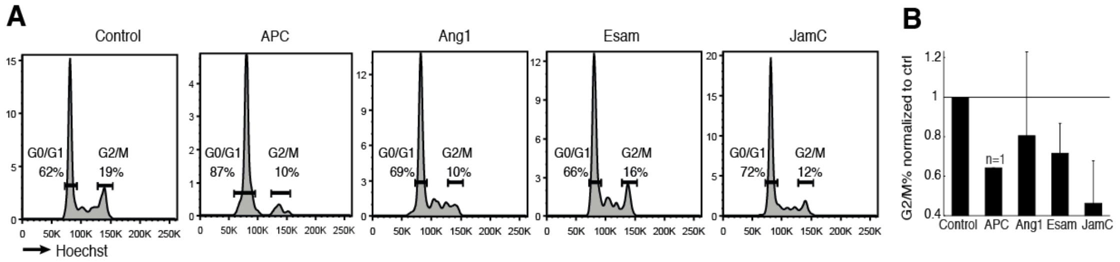

Supplementary Figure 6. HSCs exposed to an artificial in vitro niche functionalized with candidate factors show a decreased entry into cell cycle. (A) Flow cytometry histograms showing the percentage of cells in G0/G1 or G2/M phases of the cell cycle based on DNA content (Hoechst) for cells exposed to non-functionalized hydrogel (control) or hydrogel functionalized with APC, Ang1, Esam or JamC for 70 hours. (B) Percentage of cells in G2/M phase of the cell cycle for cells exposed to APC, Ang1, Esam or JamC, normalized to the percentage cells in G2/M for cells exposed to control condition. Results of two independent experiments (except for APC). Normalization performed independently for each experiment. Black bars indicate the mean. Vertical lines indicate the standard deviation.

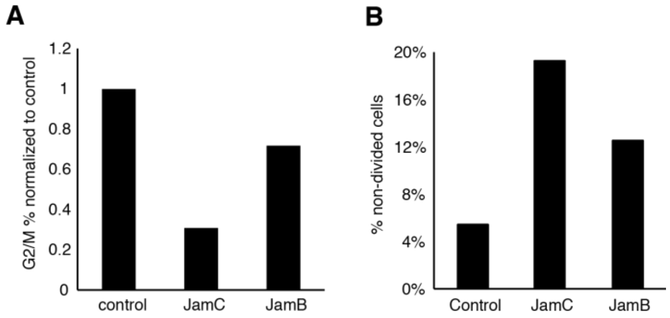

Supplementary Figure 7. HSCs exposed to JamB show reduced cell cycling, but at a lower extent than JamC. (A) Percentage of cells in G2/M phase of the cell cycle for cells exposed to JamC, or JamB, normalized to the percentage cells in G2/M for cells exposed to control condition. (B) Percentage of cells undergoing no divisions over 120 hours in non-functionalized microwells (control) or microwells functionalized with JamC or JamB. Dead cells excluded.

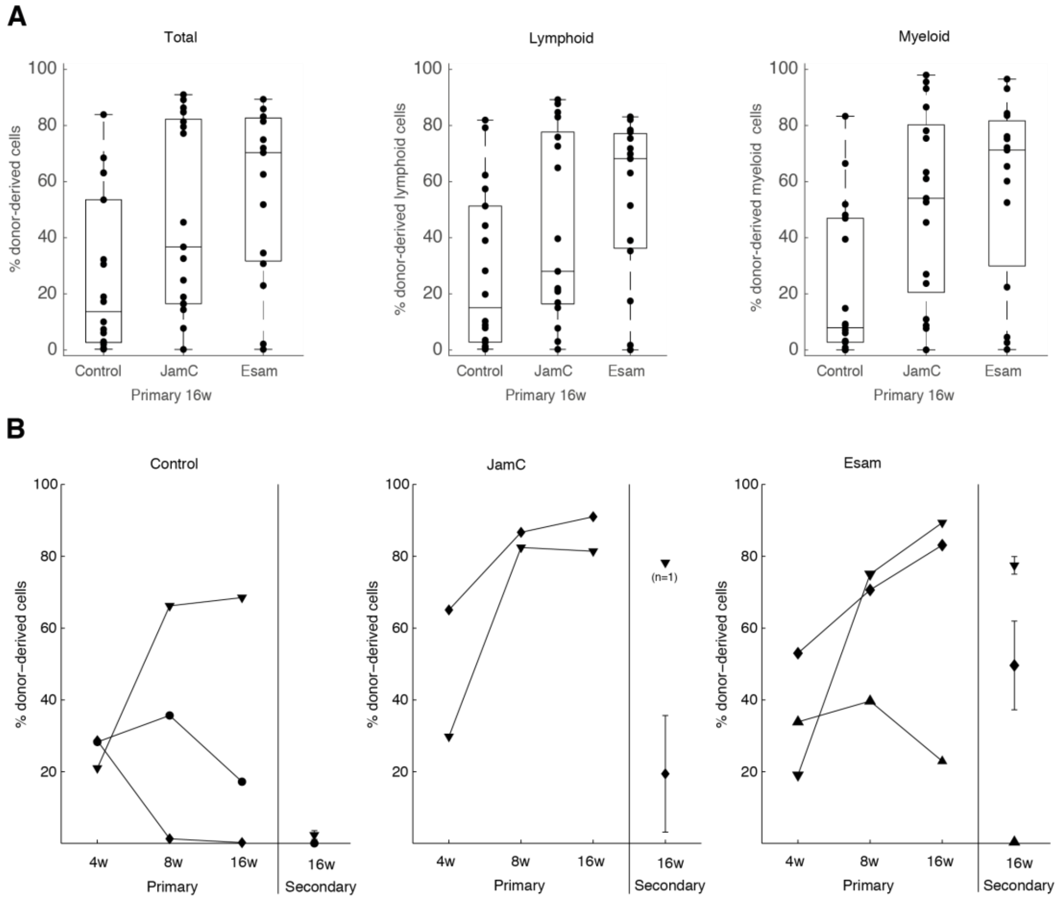

Supplementary Figure 8. Multi-lineage engraftment in primary recipients and reconstitution in secondary recipients. (A). Graph indicates percentage of donor-derived lymphoid (CD3+ CD19+) or myeloid cells (Gr1+ F4/80+) in peripheral blood at 16 weeks for primary recipients engrafted with cells exposed to non-functionalized microwells (control) or microwells functionalized with JamC or Esam. Graph represents pooled data from two independent experiments. Black dots show values for single mice and corresponding boxplots. Center lines indicate mean. Box limits indicate 25th percentile and 75th percentile. Whiskers indicate extreme data points. (B). Bone marrow was collected from primary recipients (n=3 for control, n=2 for JamC, and n=3 for Esam). Bone marrow from each donor was injected into three lethally irradiated secondary recipients at $3x10^6$ cells per secondary recipient. Blood of recipient mice was analyzed at 4 and 8 weeks. Graph indicates percentage of donor-derived cells for primary recipients at 4, 8 and 16 weeks and average percentage of donor-derived cells for the three corresponding secondary recipients at 8 weeks. Standard deviation for three secondary recipients is shown. Same symbols used for one primary recipient and its corresponding secondary recipients.

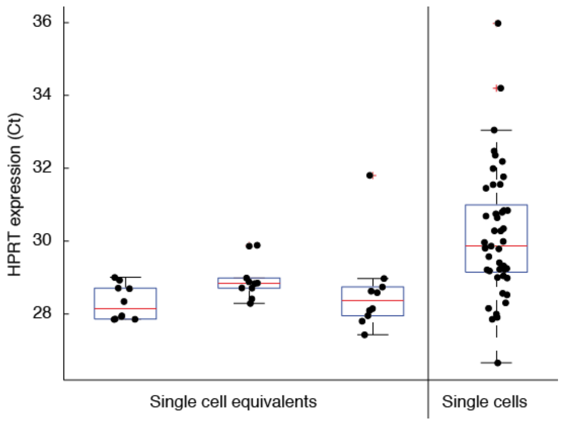

Supplementary Figure 9. Variability in housekeeping gene expression in single cells reflects biological heterogeneity. 10 single LKS CD150- cells were lysed together and the lysate was split into 10 equal volumes each representing a single cell equivalent containing the same genetic material. Graph indicates Ct values for HPRT expression for three different lysates consisting each of 10 single cell equivalents (Left, Single cell equivalents), and Ct values for HPRT expression for 45 single LKS CD150- cells lysed independently (Right, Single Cells). Black dots indicate single data points. Boxplot red center line indicate mean. Blue box limits indicate 25th percentile and 75th percentile. Whiskers indicate extreme data points excluding outliers. Red crosses indicate outliers.

Supplementary Table 1. Genes tested in the single cell multigene expression analysis.

Gene family	Gene name	TaqMan Gene Expression Assay
House keeping gene	HPRT	Mm00446968_m1
FACS markers	CD150	Mm00443316_m1
	CD48	Mm00455932_m1
	CD34	Mm00519283_m1
ECM	Tgm2	Mm00436987_m1
	Bgn	Mm00455918_m1
Adhesion	Esam	Mm00518378_m1
	C-kit	Mm00445212_m1
	Tie2	Mm00443243_m1
	Jam3	Mm00499214_m1
Intracellular signaling	Grb10	Mm01180443_m1
	ProCR	Mm00440992_m1
	Fhl1	Mm03009774_m1
	b-cat	Mm00483039_m1
Cell cycle	P57	Mm00438170_m1
	P27	Mm00438168_m1
	P21	Mm00432448_m1
	P130	Mm00618533_m1
	Pten	Mm00477208_m1
Transcription factors	Pbx1	Mm01701537_m1
	Gata3	Mm00484683_m1
	C-myc	Mm00487803_m1
	Hoxb4	Mm00657964_m1
DNA repair	Gadd45	Mm00432802_m1
